# Laboratory Assessment of the Effects of AGA@4life Multidisciplinary Intervention on the Inflammatory Profile, MMPs, and TIMPs in a Geriatric Population

**DOI:** 10.3390/healthcare12050509

**Published:** 2024-02-21

**Authors:** Ana Patrícia Lourenço, Catarina Freitas, Maria Helena Timóteo, Maria Soares, João Paulo Figueiredo, Nádia Osório, Ana Valado, Maria Trapali, Telmo Pereira, Armando Caseiro

**Affiliations:** 1 Polytechnic Institute of Coimbra, Coimbra Health School, Biomedical Laboratory Sciences, Rua 5 de Outubro, S. Martinho do Bispo, 3046-854 Coimbra, Portugal; catarina.freitas@medisyn.ch (C.F.); uc2022159646@student.uc.pt (M.S.); nadia.osorio@estesc.ipc.pt (N.O.); valado@estesc.ipc.pt (A.V.); armandocaseiro@estesc.ipc.pt (A.C.); 2 Polytechnic Institute of Coimbra, Coimbra Health School, Social and Human Sciences, Rua 5 de Outubro, S. Martinho do Bispo, 3046-854 Coimbra, Portugal; jpfigueiredo@estesc.ipc.pt; 3 LABINSAÚDE—Research Laboratory for Applied Health Sciences, Polytechnic Institute of Coimbra, Coimbra Health School, Rua 5 de Outubro, S. Martinho do Bispo, 3046-854 Coimbra, Portugal; telmo@estesc.ipc.pt; 4 Laboratory of Chemistry, Biochemistry and Cosmetic Science, Department of Biomedical Medicine, University of West Attica, Ag. Spyridonos Str., 12243 Egaleo, Greece; ymaria@uniwa.gr; 5 Polytechnic Institute of Coimbra, Coimbra Health School, Clinical Physiology, Rua 5 de Outubro, S. Martinho do Bispo, 3046-854 Coimbra, Portugal

**Keywords:** aging, IL-6, TNF-α, MMPs, TIMPs

## Abstract

In recent years, the world’s aging population has increased, contributing to the development of age-related pathologies, which have been aggravated by physical inactivity and excessive fat intake. This study aimed to evaluate the effect of implementing a nutritional program (control group—CG) combined with exercise (intervention group—IG) on the inflammatory profile, MMPs, and TIMPs in a group of 34 elderly participants (IG, *n* = 18; CG, *n* = 16). Participants underwent a full multidisciplinary diagnostic evaluation (T0), with the gathering of clinical information and biochemical and hematological determinations being re-evaluated eight weeks later (T1). A diet manual was made, which provided a selection of different types of diets resulting from the nutritional needs of the different users at the center. The aerobic exercise consisted of two sessions per week with a total duration of 1 h. The laboratory evaluation was performed by slot blot. Statistical analysis included a paired sample *t*-test and Spearman’s correlation coefficient. We observed that in the IG, there was a significant increase at T1 of TNF-α (*p* < 0.05) and MMP-2 (*p* < 0.05), without changes in IL-6 and MMP-9, showing that the intervention did not cause an exacerbated inflammatory response in exercised elderly people. The intervention program implemented showed potential to contribute to better active aging strategies, taking advantage of the known benefits of exercise without inducing a harmful inflammatory response in elderly participants.

## 1. Introduction

Increased average life expectancy and declining birth rates have led to an exponential rise in the aging of populations, becoming a major social and economic challenge worldwide, namely, the inclusion of elderly people in society, their active life expectancy, their quality of life, and the sustainability of health and social security [1,2].

Old age remains the largest risk factor for most chronic diseases, such as cardiovascular diseases, cancer, stroke, chronic obstructive pulmonary disease, chronic kidney disease, type 2 diabetes, and Alzheimer’s, and is responsible for increased morbidity, hospitalizations, and mortality, which leads to a significant increase in health and social costs [1,2,3]. According to the United Nations, the over-60 population is growing by about 3% a year [4]. In 2019, 9% of the world’s population and 19% of the European population were over 65 [5]. In the same year, the elderly population in Portugal was 22.1% [6].

Physiological decline leads to increased chronic inflammation (CI), oxidative stress, DNA damage, mitochondrial function decline, and cellular senescence, contributing to the onset of metabolic syndrome (MS) and age-related disorders. CI can persist over long periods of time, generating irreversible organ dysfunction due to tissue damage and/or fibrogenesis [1,3,7]. In addition, the lack of daily physical activity (PA) and a high-fat diet further aggravate the onset of certain changes, such as obesity [1,3,7]. According to a study conducted in 2018, 44% of Portuguese elderly were overweight, and 39% were obese [8]. Thus, it is essential that there is a multidisciplinary intervention in the lifestyle of the elderly population in order to improve their well-being, health, and quality of life [1,3,7].

Advanced age represents a risk factor for reduced mobility that, associated with changes in body composition, may have adverse effects [9,10]. The prevalence of sarcopenia and obesity tends to increase, and these conditions are an important cause of frailty, CI, and old age inability and dependence [9,10,11,12].

Sarcopenia is a major public health problem that affects older people and is associated with a bad quality of life [13,14]. It is an age-dependent geriatric syndrome characterized by a progressive, generalized skeletal muscle disorder that is associated with physical disability, metabolic dysfunction, and increased mortality [13,15]. Elderly people with sarcopenia have a very high risk of falls, fractures, pulmonary insufficiency, sleep disorders, cognitive deficit, disability, hospitalization, and premature mortality [13,14]. Since there is no specific treatment, exercise is the most effective intervention method for sarcopenia as a prevention and primary treatment, which could reduce the health costs associated with this disease over time. However, the best types of exercise have not yet been established [13,14].

In an easy and understandable way, obesity is defined as an excessive increase in fat mass. Its prevalence in Europe has increased, and the number of overweight people is growing at an alarming rate [15]. It affects morbidity, disability, and daily activities and increases the risk of developing cardiovascular diseases such as heart disease and strokes, type 2 diabetes, musculoskeletal disorders like osteoarthritis, some cancers, and other health-related problems [15].

The Mediterranean diet (MD), indicated as a healthy eating model, contributes to a better biochemical profile and improved quality of life [16]. It is based on a high proportion of monounsaturated dietary lipids compared to saturated ones (mostly due to the consumption of olive oil); high intake of vegetables, fruit, pulses, nuts, and unrefined cereals (bread included); reduced consumption of meat and meat products; moderate intake of milk and its derivatives; aromatic herbs used instead of salt; water as the main drink; and moderate intake of alcohol, especially wine, at main meals [16]. Fish is also included, but it is dependent on proximity to the sea. It is associated with several health benefits, such as reducing the risk of cardiac pathologies, obesity, and diabetes, among others. A published study indicated that 43.7% of the Portuguese elderly had an MD; however, about 21% of the food ingested was not represented in the food wheel [16].

It is estimated that physical inactivity is the fourth largest risk factor for global mortality, causing about 3 million deaths per year. According to European data, elderly people spend an average of 9.4 h per day in sedentary activities, and only 7% have regular PA [17].

With increasing age, the way the body digests food, absorbs nutrients, and stores energy changes [10]. In this sense, the regular practice of PA provides several health benefits, such as increased immune function and muscle mass and decreased amount of adipose tissue, important factors for the prevention/improvement of MS, diseases related to aging, and decreased/suppression of CI, resulting in a better quality of life for elderly people [3,7,10,13,15,17,18,19,20,21,22].

Changes related to physiological decline have been described in all organs and tissues, including the immune system. These changes affect innate and acquired immunity and are related to immune senescence [3]. They are accompanied by a persistent pro-inflammatory state, with a high presence of pro-inflammatory cytokines, such as interleukin-6 (IL-6) and tumor necrosis factor-alpha (TNF-α), which are excessively secreted by inflammatory cells, leading to the development of various pathologies that negatively affect people’s healthy life expectancy [7,23,24].

On the other hand, a high-fat diet and a sedentary lifestyle contribute to the accumulation of adipose tissue, which also contributes to the infiltration of inflammatory cells and induces the prolonged production of IL-6 and TNF-α that, in excess, will induce skeletal muscle atrophy [7,18,24]. By increasing the demand for energy, thus reducing the size of adipocytes and, therefore, the number of immune cells in this tissue, PA has an anti-inflammatory effect. This means that by reducing the levels of these cytokines, the inflammatory status of elderly people will be changed, leading to a decrease in CI [3,7,17,22]. Studies have shown that 12 weeks of PA allowed lower levels of TNF-α and that it caused insulin resistance [18,19]. It also has a central role in autoimmune diseases and is elevated when there is adipose tissue hypertrophy [20]. Muscle contraction and increased energy metabolism will lead to the production of cytokines called myokines [3,7]. IL-6 induced by PA is one of the main contributors to its anti-inflammatory effect [7]. It is involved in glucose and lipid metabolism and stimulates cortisol production and anti-inflammatory cytokines that will inhibit TNF-α synthesis. During PA, IL-6 is the first cytokine to be detectable in plasma, and the longer the duration and intensity of PA, the more pronounced its systemic response will be [18,20].

A group of enzymes called matrix metalloproteinases (MMPs) and their endogenous tissue inhibitors (TIMPs) regulate the homeostasis of the extracellular matrix (ECM). When there is an imbalance in the turnover of ECM components during aging, this may result in unfavorable remodeling processes and change the activity of MMPs [25,26].

MMPs are calcium and zinc-dependent endopeptidases that regulate the composition of ECM. They are produced by different types of cells, can degrade various ECM components, and may be described as biological markers of response to inflammatory processes [25,26,27,28]. There are more than 25 MMPs identified in humans, which are divided into six subgroups [29,30]. In the gelatinase subgroup, we have MMP-2 and MMP-9, also known as gelatinases A and B, respectively, which are associated with the practice of PA and obesity [27,31]. It was recently demonstrated that the activity of these MMPs in blood circulation may be considered a mediator of inflammation, providing relevant information on the inflammatory status during the aging process and in pathological conditions [26,32]. Several studies indicate that the practice of PA (without muscle damage) is associated with a reduction in the levels of these MMPs in blood [27,28,31,32,33].

Tissue inhibitors of metalloproteinase (TIMPs) regulate the activity of MMPs. This family consists of four members: TIMP-1, TIMP-2, TIMP-3, and TIMP-4. In general, all TIMPs competitively and reversibly inhibit MMP activity. However, TIMP-1 is closely related to MMP-9, whereas TIMP-2 binds strongly to MMP-2. An alteration in the MMP/TIMP balance may result in the appearance of several pathologies [27,32,33].

In conclusion, this project aimed to understand the effects of PA and a balanced diet on the inflammatory profile, MMPs, and TIMPs in the elderly population under study, contributing to promoting active aging strategies and improving the quality of life of elderly people.

## 2. Materials and Methods

### 2.1. Study Sample

To carry through AGA@4life program, a multidisciplinary team was involved, including a physiotherapist, a dietitian, a physiologist, psychologists, audiologists, and biomedical laboratory scientists [1].

This program consisted of six main steps: 1—data gathering through a diagnostic evaluation of the participants. 2—Discussion of cases between the multidisciplinary working group. 3—Definition of an individualized and multidisciplinary intervention plan. 4—Implementation of the intervention plan together with elderly people, family members, and caregivers. 5—Monitoring the intervention plan response. 6—Revising the intervention according to the outcome [1].

In this study, 34 elderly people from a day care center in Coimbra district, Portugal, participated in this study; they were Caucasians, aged 83.3 ± 6.9 years, of both sexes, physically autonomous, and had no previous history of cerebrovascular or neurological diseases [1].

Participants underwent an initial multidisciplinary diagnostic evaluation (T0), with collection of clinical and demographic information, namely data on comorbidities, diet, PA and cardiovascular risk profile, treatments, functional capacity and disorders, and biochemical and hematological determinations [1]. The generality of participants had hypertension and dyslipidemia.

For determination of blood parameters, 10 mL of peripheral blood was collected by venipuncture to obtain serum and plasma samples (K_3_EDTA—ethylenediaminetetraacetic acid tripotassium) [1]. Samples were centrifuged for 10 min at 3500× *g*, and serum was stored at −70 °C until analysis [34]. A complete blood count was performed using the hematologic analyzer Cell-Dyn Sapphire^TM^ (Abbott Laboratories, Santa Clara, CA, USA). The general biochemical profile included the following parameters: albumin, total proteins, total bilirubin, total cholesterol, HDL cholesterol, LDL cholesterol, glucose, creatinine, uric acid, urea, triglycerides, alanine aminotransferase, aspartate aminotransferase, amylase, lactate dehydrogenase, alkaline phosphatase, gamma-glutamyltransferase, creatine kinase, calcium, C-reactive protein. Biochemical parameters were determined by spectrophotometry in the Prestige 24i autoanalyzer (Tokyo Boeky, Tokyo, Japan) with Cormay^®^ reagents (Warsaw, Poland). All assays were validated and passed quality control [1].

For the nutritional intervention at T0 and after the implementation of the program, the Mini Nutritional Assessment (MNA) questionnaire, validated in Portugal, was used to determine the nutritional risk; Food Frequency Questionnaire, validated in Portugal, was used to establish the food intake and frequency history; and 24 h assessment was used to determine the food intake in that period [1]. Anthropometric data (AD) were obtained, including height, weight, body mass index, waist/hip ratio, and waist, arm, and leg circumference, using a non-stretchable tape measure. The percentage of fat and lean mass was determined by bioelectrical impedance analysis. The first phase, through MNA and AD, allowed the nutritional needs to be estimated and an individualized diet plan to be drawn up, considering the clinical history, anamnesis, and biochemical parameters of each participant [1]. The food plan was given and explained to each day center user. This intervention also included an audit during the preparation of the meals, and, in accordance with the results obtained, training was given to the people responsible for preparing the daily meals. During the training, topics such as healthy eating and the specific nutritional needs of elderly people were discussed [1]. Recommendations were also given to correct the main dietary errors observed. A diet manual was made, which provided a selection of different types of diets, programmed and typified, with an indication of forbidden and permitted foods and dishes resulting from the nutritional needs of the different users at the center [1].

The participants in the exercise program (EP) were divided, according to their willingness, into control group (CG, *n* = 16) without EP intervention and intervention group (IG, *n* = 18) with outdoor aerobic exercise sessions [1]. After T0, CG kept their daily routines while the IG was submitted to a plan based on tailored aerobic exercise sessions and on OTAGO incorporated in a technological system (FallSensing) with pressure and inertial sensors, feedback, and Exergames for 8 weeks, 3 times a week, lasting approximately 20 min [1].

The exercise program focused mainly on the flexors and extensors muscles of the knee, hip abductors, ankle dorsi flexor/plantar flexor muscles, and balance. According to the recommendations of the World Health Organization, aerobic exercise sessions consisted of two sessions per week with a 10min walk on flat or slightly sloping ground, balance and coordination exercises, and joint mobilization exercises with respiratory coordination, with a total duration of 1 h [1].

At T1, the evaluations carried out at T0 were repeated for the participants who completed the EP. The CG and IG were composed of 7 and 11 participants, respectively, being the dropouts justified by hospital internments and exchange of day center [1].

### 2.2. Evaluation of IL-6, TNF-α, MMP-2, MMP-9, TIMP-1, and TIMP-2 Serum Levels

To evaluate the inflammatory response through IL-6 and TNF-α levels, as well as the impact of MMPs and TIMPs, both in the CG and GI, serum determination was performed by slot blot. This technique was performed as described in Caseiro et al. [35].

Firstly, two dilutions were performed to ensure the quantity of proteins present in the different samples was uniform. Subsequently, nitrocellulose membranes (Whatman^®^, Protan^®^, Merck Life Science, Darmstadt, Germany) were activated with 10% methanol, which remained in 10% TBS (Tris-buffered saline) after washing in distilled water. The samples were then applied to the membranes using a 24-well microfiltration device connected to a vacuum pump.

After drying, the membranes were blocked with 5% (*w*/*v*) dry non-fat milk dissolved in 100 mL of 0.1% TBS-T (Tris-buffered saline tween) for 1 h. Then, the membranes were washed three times with TBS-T, each time for 10 min.

After that, 10 µL of primary antibody (Ab) (1:1000) was diluted in 10 mL of blocking solution. The membranes were incubated with the respective Ab: anti-IL-6 (Clone 6708, MAB206, R&D Systems, Abingdon, UK), anti-TNF-α (Clone 28401, MAB610, R&D Systems, Abingdon, UK), anti-MMP-9 (Clone 36020, MAB936, R&D Systems, Minneapolis, MN, USA), anti-MMP-2 (Clone 101721, R&D systems, Minneapolis, MN, USA), anti-TIMP-1 (clone 63515, R&D Systems, Abingdon, UK), or anti-TIMP-2 (clone 127711, R&D Systems, Abingdon, UK). After incubation for 3 h under constant stirring, the membranes were washed twice with TBS-T for 10 min each.

Subsequently, the membranes were incubated with secondary Ab (horseradish-conjugated anti-mouse, GE Healthcare, Cleveland, OH, USA) for 3 h (1:1000; 10 µL of Ab diluted in 10 mL of blocking solution) under constant stirring. After the incubation period, the membranes were washed twice with TBS-T for 10 min each. The substrate was placed immediately before film development.

Detection was performed by chemiluminescence, according to the manufacturer’s instructions (GE Healthcare, Cleveland, OH, USA), through exposure to Kodak BioMax Light Film (Carestream Health, Rochester, NY, USA) on a Kodak^®^ X-OMAT Cassette (Carestream Health, Rochester, NY, USA) for 15 min. Film images were achieved using the GelDoc XR system (Bio-Rad, Hercules, CA, USA), and quantitative analysis of optical density was performed with Quantity One^®^ 1-D analysis software, version 4.6.6 (Bio-Rad, Hercules, CA, USA) [34].

### 2.3. Statistical Analysis

To evaluate the effects of the implementation of a nutritional program and exercise sessions on the inflammatory profile, MMPs, and TIMPs of the study participants, statistical analysis and interpretation of the results were performed. For this prospective study (two time points), considering the outcome IL-6 levels in serum and an effect size d ≈ 0.54, a sample of 30 participants was estimated and given a test power of 0.8 (1-β), a confidence level of 95%, and a predicted sampling error of 5% (α < 0.05).

To describe the distribution of sample values of the groups previously identified, we used a descriptive univariate statistic. Descriptive statistical measures applied were measures of central tendency, complemented by measures of dispersion and quantiles. As support for a better understanding of statistical information, graphical analysis was also used, such as bar diagrams, extreme and quartile diagrams, and scatter diagrams.

For the statistical decision, a set of assumptions regarding the shape measures for the distribution of quantitative parameters were fulfilled. Considering that the study sample consisted of 18 individuals, data normality was assessed by the Shapiro–Wilk test (*n* ≤ 50). Regarding the evaluation of frequency distribution, concerning symmetry, standardized values of the Z statistic (Skewness coefficient/Skewness Std error) were used, and for flatness, this type of evaluation was also obtained using standardized values of the Z statistic (Kurtosis coefficient/Kurtosis Std error). The distribution tends to be symmetric and mesokurtic because the modulus result was less than or equal to 1.96.

Based on the information above, parametric paired-sample *t*-test was used to evaluate internal variations within each group (univariate analysis), and parametric independent sample *t*-test was used to evaluate differences between groups (univariate analysis). To analyze the correlation between the obtained parameters, Spearman’s correlation coefficient test was applied (bivariate analysis).

The results presented are expressed in arbitrary units (AU). For statistical inference, we had a confidence level of 95% for a maximum random error of up to 5% inclusivity. Statistical treatment of data was performed using the IBM SPSS^®^ v.27 software (National Opinion Research Center, Chicago, IL, USA).

## 3. Results

### 3.1. Evaluation of Serum Levels in the Control Group

Data were analyzed using Student’s *t*-test for paired samples, and it was found that, for the parameters assessed in the CG, there were no statistically significant differences between the serum values obtained at the two times of assessment (*p* > 0.05; Table 1).

Regarding IL-6 (t = 1.309; df = 5), we found that the results obtained were not statistically significant (*p* = 0.247). Therefore, although we observed a slight decrease between T0 ($\bar{x}$ = 0.928 ± 0.228 AU) and T1 ($\bar{x}$ = 0.892 ± 0.204 AU), the mean difference (MD) obtained was not statistically significant (MD = 0.037 AU). Moreover, we can also see that from the total of individuals evaluated (6), 50% (3) had decreased IL-6 serum values, 33% (2) had an increase in this value, and only 17% (1) remained the same between T0 and T1.

As for TNF-α (t = 1.184; df = 6), a reduction in the serum values obtained after the implementation of the nutritional program ($\bar{x}$ = 1.957 ± 1.318 AU) compared to the initial assessment ($\bar{x}$ = 2.399 ± 1.192 AU) was observed. However, the mean difference obtained (MD = 0.441 UA) did not prove to be statistically significant (*p* = 0.281). Of the individuals evaluated in this group (7), 57% (4) showed lower serum values after the implementation of the nutritional program. Contrarily, 43% (3) showed an increase in this value at the second time of evaluation.

Concerning MMP-2 (t = 0.661; df = 6), we found a slight decrease between T0 ($\bar{x}$ = 2.399 ± 1.192 AU) and T1 ($\bar{x}$ = 0.784 ± 0.273 AU), which was not shown to be statistically significant (*p* = 0.533). The mean difference between them was 0.026 AU. Of all assessed individuals in the CG (7), we found that the percentage of people in whom there was a tendency to increase or decrease serum levels of MMP-2 between the two times of assessment was equal (43%). However, in 14% (1) of the individuals, no changes were observed between T0 and T1.

For MMP-9 (t = −1.750; df = 6), there was a slight increase in the values at T1 ($\bar{x}$ = 0.784 ± 0.273 AU) when compared to T0 ($\bar{x}$ = 0.937 ± 0.447 AU), and the mean difference (MD = −0.220 AU) did not prove to be statistically significant (*p* = 0.131). We can also add that from the totality of individuals assessed (7), only 29% (2) had decreased serum levels at T1, and 71% (5) had increased values at the same time of assessment.

Regarding TIMP-1 (t = −1.829; df = 6), we observed a slight increase after the nutrition program implementation ($\bar{x}$ = 1.457 ± 0.436 AU) compared to the baseline period ($\bar{x}$ = 1.289 ± 0.543 AU). This resulted in a mean difference of −0.168 AU, which was not proved to be statistically significant (*p* = 0.117). Of the total individuals evaluated in this group (7), only 14% (1) showed a decrease in serum values between T0 and T1. On the other hand, 86% (6) exhibited higher amounts of TIMP-1 at T1.

Finally, for TIMP-2 (t = 1.358; df = 6), we found that the differences observed were not statistically significant (*p* = 0.223). Still, we observed a weak decrease between T0 ($\bar{x}$ = 0.603 ± 0.403 AU) and T1 ($\bar{x}$ = 0.537 ± 0.341 AU), with a mean difference of 0.066 AU. Of all the individuals evaluated (7), 57% (4) had decreased serum values between the two assessment times, and 43% (3) increased the value.

### 3.2. Evaluation of Serum Levels in the Intervention Group

Applying the Student’s *t*-test for paired samples, statistically significant differences were observed for IG in the serum concentration of TNF-α and MMP-2 between the two times of assessment (*p* < 0.05). This indicates that the practice of PA significantly influenced the serum levels of these parameters. However, there were no statistically significant differences in serum levels of IL-6, MMP-9, TIMP-1, and TIMP-2 since the *p* was >0.05 (Table 2).

Regarding TNF-α (t = −3.226; df = 10), we verified that there was an increase in serum levels after the implementation of EP ($\bar{x}$ = 2.361 ± 0.450 AU) compared to the initial assessment ($\bar{x}$ = 1.956 ± 0.458 AU). This resulted in a mean difference of −0.404 AU, which was shown to be statistically significant (*p* = 0.009). Of the total of individuals assessed (11), only in 18% (2) did we find a decrease in values between the times of assessment. Nevertheless, 82% (9) showed increased levels at the second time of assessment.

As for the MMP-2 (t = −2.467; df = 10), the results obtained were also statistically significant (*p* = 0.033). There was an increase in serum values for this parameter between T0 ($\bar{x}$ = 0.562 ± 0.092 AU) and T1 ($\bar{x}$ = 0.649 ± 0.096 AU), with a mean difference of −0.087 AU. Therefore, of all the individuals assessed in this group (11), only 18% (2) showed a lower value at T1; on the contrary, 72% (8) increased their serum levels at the same time of assessment. Still, 9% (1) maintained the same serum levels between the two assessments.

For the IL-6 parameter (t = −1.232; df = 10), we observed a slight increase in its quantity at T1 ($\bar{x}$ = 0.673 ± 0.115 AU) when compared to T0 ($\bar{x}$ = 0.641 ± 0.129 AU), but this mean difference (M = −0.032 AU) was not statistically significant (*p* = 0.246). We should also add that, of all the individuals assessed (11), 45.5% showed an increase (5) and decrease (5) in serum levels between T0 and T1. However, 9% (1) remained equal between the two times of evaluation.

Concerning MMP-9 (t = −0.762; df = 10), we found a small increase in its serum concentration between T0 ($\bar{x}$ = 0.700 ± 0.213 AU) and T1 ($\bar{x}$ = 0.726 ± 0.217 AU); however, there was no statistical significance (*p* = 0.464). The mean difference between the two instants was −0.026 AU. We may also mention that, from the total number of individuals evaluated in this group (11), only 36% (4) had decreased levels at T1, and the remaining 64% (7) increased their values after the implementation of the EP.

For TIMP-1 (t = 0.312; df = 10), there was a slight decrease between the first ($\bar{x}$ = 1.159 ± 0.230 AU) and second times of evaluation ($\bar{x}$ = 1.137 ± 0.142 AU), but the mean difference (MD = 0.022 AU) was not statistically significant (*p* = 0.761). Considering all the individuals assessed (11), 45.5% (5) had decreased and 54.5% (6) had increased serum levels at the second time of assessment.

Finally, with respect to TIMP-2 (t = −0.062; df = 10), after performing the EP ($\bar{x}$ = 0.719 ± 0.085 AU), we observed a subtle increase in the measured quantity compared to the initial assessment ($\bar{x}$ = 0.717 ± 0.142 AU). Thus, the mean difference obtained (MD = −0.002 AU) showed no statistical significance (*p* = 0.952). Of all individuals assessed in GI (11), there was, in percentage terms, equality in the increase and decrease in serum levels for this parameter at T1, both of 45.5% (5). On the other hand, 9% (1) of the individuals maintained the values at both times of assessment.

### 3.3. Correlation between Parameters

Spearman’s rank correlation coefficient test was used to check the correlations between the parameters assessed in IG in the T0 and T1 assessments. (Table 3).

In IG, when IL-6 and TNF-α were correlated at T0 (rho = −0.182; *p* = 0.593), we found a low negative correlation and no statistical significance between these parameters. However, we confirmed that at T1, this correlation was moderately positive (r = 0.337; *p* = 0.311).

**Figure 1:**
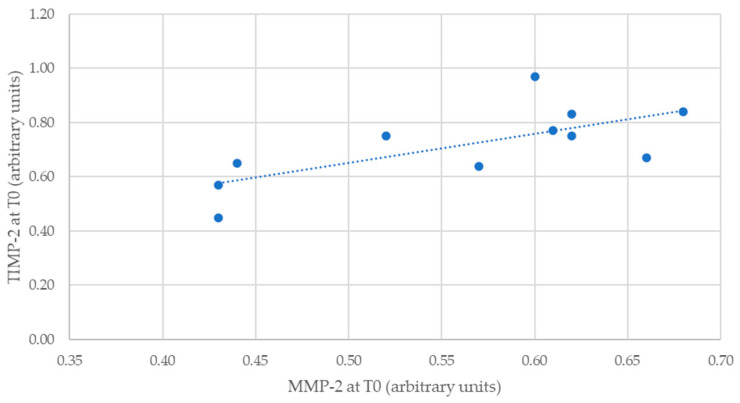
Correlation between MMP-2 and TIMP-2 levels at T0 for the intervention group.

Considering MMP-2 and TIMP-2 levels, it was possible to verify an inverse correlation between the two times of evaluation. If, at T0 (rho = 0.689; *p* = 0.019), it was moderately positive and statistically significant (Figure 1), at T1 (rho = −0.402; *p* = 0.221), it was moderately negative and not statistically significant.

**Figure 2:**
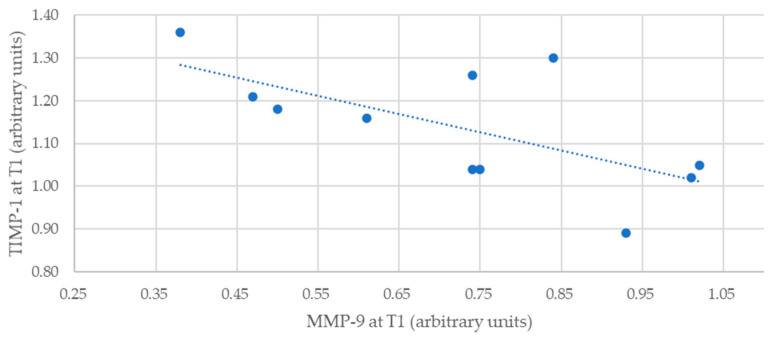
Correlation between MMP-9 and TIMP-1 levels at T1 for the intervention group.

Regarding MMP-9 and TIMP-1, we observed that the correlation for T0 was negative and low (rho = −0.336) and without statistical significance (*p* = 0.312). However, the values at T1 (rho = −0.610; *p* = 0.046) showed a moderately negative and statistically significant correlation (Figure 2).

## 4. Discussion

In this study, we aimed to evaluate possible changes in the inflammatory profile, MMPs, and TIMPs in a group of elderly people before and after the establishment of a nutritional and exercise program. To this end, we divided the sample into CG (without EP) and IG (with EP). Both groups benefited from a nutritional plan, which was adjusted to each participant.

As for the CG, it did not show statistically significant changes in the serum values of the parameters analyzed between the two times of evaluation. This means that, by itself, the implementation of a nutritional program was not enough to cause relevant changes in the concentrations of the parameters studied. However, the serum levels evaluated at T0 were, on average, higher than those obtained at T1 after the implementation of the nutritional program, except for MMP-9 and TIMP-1. This is according to what is expected since a healthy and adjusted nutrition is partly responsible for the reduction in the amount of adipose tissue [1,7,16,18,24]. It is known that adipocyte hypertrophy and hyperplasia and increased pro-inflammatory cytokines, such as IL-6 and TNF-α, are strongly related to obesity, which is one of the causative agents of chronic inflammation [7,10,18,20,24,36]. Both IL-6 and TNF-α are commonly used as markers in dynamic inflammatory responses to high-fat meals [24]. On the other hand, higher levels of MMPs are associated with obesity [31]. There is not much information on the relationship of MMPs with diet, nor a very comprehensive understanding of the relationship between them and obesity; however, it is known that adipocytes play an important role in the production and release of MMP-2 and MMP-9 in individuals with obesity and that they are elevated in these individuals [28,31]. On the other hand, the state of inflammation associated with obesity and physiological decline may be responsible for the chronic increase in the activity of gelatinases, as compared to healthy individuals [28,33].

In IG, we observed a statistically significant increase in TNF-α and MMP-2, with the IL-6 and MMP-9 values being similar between the two assessments. Therefore, we verified that the implementation of this exercise program in older people did not trigger an exacerbated inflammatory response, with potential risk for participants, keeping the values of the pro-inflammatory cytokine IL-6 and MMP-9 stable. PA has an anti-inflammatory effect on the body, but in specific populations, namely aged people, a contrary response could be observed [3,17,18,19,20,22,28,36,37]. Exercise sessions could initiate a complex cascade of inflammatory events dependent on the type, intensity, duration, and familiarity of the exercise, as well as the age and clinical condition of the study subjects [3,20,37]. In this way, the results observed for TNF-α and MMP-2-d are acceptable since people with some age, chronic inflammation, and obesity showed high levels of the parameters evaluated when compared to healthy individuals [18,20,28,33].

Regarding the inflammatory profile, it is known that the levels of IL-6 and TNF-α tend to decrease after adequate and ideal exercise [17,18,20,22,24,28]. Some studies suggested that moderate acute increases in IL-6, caused by PA, exert direct anti-inflammatory effects through inhibition of TNF-α [3,20,36]. However, the production of IL-6 during exercise has been described as “a double-edged sword” due to its beneficial and harmful effects (3). On the one hand, it exerts an effect on TNF-α inhibition, resulting in protection against chronic diseases associated with inflammation; on the other hand, it may initiate an acute phase inflammatory response to exercise (with TNF- α release). The above is sufficient to induce trauma in muscle fibers since the inflammatory process is essential for muscle repair to occur [3,28,37]. Studies have reported that TNF-α and mainly IL-6 increased with a moderate-intensity exercise session and increased even more with an intense exercise session [3,18,37]. The greater the intensity and the quantity of muscles involved, the greater the concentrations of IL-6 obtained [3,20]. In addition, exercise duration was responsible for more than 50% of the variation in circulating concentrations of muscle-derived IL-6 [3,20]. However, it is also known that, despite the increase in these parameters, their concentrations are not linear over time, eventually decreasing as time goes by [20,28,37]. This situation becomes beneficial since the excess IL-6 and TNF-α induces skeletal muscle atrophy, which results in reduced strength and muscle function, as well as increased muscle pain [18,37]. Moderate and regular PA helps in the maintenance of optimal levels of IL-6 (anti-inflammatory effect), resulting in an improvement in health, which reinforces the importance of exercise in the daily routines of elderly people with low levels of strength and a sedentary lifestyle [18,20,22,25,28].

Compared to what was said for the inflammatory profile, the response of gelatinases to PA also depends on the mode, duration, and intensity of the exercise performed [25,28,31,33]. Considering that TIMPs occur simultaneously (in a 1:1 ratio), we can say that their serum concentration depends on the same factors as MMPs [27]. The proteolytic activity of MMPs is normally reduced in healthy and undamaged tissues. Nevertheless, lesions in skeletal muscle induced by exercise increase the degradation of ECM, which translates into increased expression, as well as an increase in its inhibitors [25,27,32]. It is known that both MMP-2 and MMP-9 are involved not only in inflammatory responses but also in ECM remodeling and muscle regeneration [27]. MMP-2 can be activated simultaneously with muscle fiber regeneration, while MMP-9 can be expressed in association with early inflammatory response and satellite cell activation [27,33]. On the other hand, regarding TIMPs, it is known that TIMP-1 is activated during the early phase of muscle damage, while TIMP-2 is expressed during the later stages of muscle damage [27]. In several studies carried out with different samples, we found that the variations for these parameters were not consensual. For both MMP-2 and MMP-9, we found that there could be an increase in their values, especially if the PA was of higher intensity [27,28,31,33]. The same was found for TIMP-1 and TIMP-2, in which exercise intensity proved to be directly proportional to increased concentrations [25,27,31,33]. It should be noted that these changes were dependent on the time of evaluation, as their concentrations tended to decrease over time [25,27,28,31,33]. The expression of MMPs is positively regulated by several inflammatory cytokines, including TNF-α, and MMPs can cleave and activate TNF-α [32]. In this sense, the increase of both corroborates what has already been described. As moderate and regular PA practice has already demonstrated an anti-inflammatory effect, decreasing the concentrations of gelatinases and, subsequently, of their inhibitors, it is important that this be part of the daily routine of elderly people [31,33].

Still, regarding the IG, it was interesting to analyze the inverse direction of the correlation between IL-6 and TNF-α and between MMP-2 and TIMP-2 at the two times of evaluation. If, at T0, there was a low negative correlation for the inflammatory parameters, at T1, it was moderately positive, which may be related to the changes introduced. On the other hand, between MMP-2 and TIMP-2, the correlation was statistically significant, positive, and moderate at T0. On the contrary, at T1, the relationship between them was moderate, negative, and without statistical significance. Considering that these parameters are closely related, the results obtained at T1 do not go against what we expected at the beginning. We also observed a significant negative and moderate correlation between MMP-9 and TIMP-1 for the IG at the second time of evaluation. In this regard, the increase in MMP-9 was inversely proportional to the increase in TIMP-1.

After the previous explanation, we considered that the results that were not in agreement with what we had initially predicted might have occurred because of several factors. Among them, we highlight the moment when we collected the blood for analysis, which may have contributed to the high concentrations we found in the different parameters, as samples collected immediately after exercise cannot rule out the effects of dehydration on plasma volume and, consequently, the quantification of the inflammatory markers measured [37]. Another explanation lies in the 8 weeks of exercise since they may not have been enough for us to detect significant decreases in serum levels. Longer follow-ups could provide more information about the effects of the interventions and their long-term impact on the inflammatory profile. Regarding sample size, the larger the sample, the more robust and generalizable the results could be. Also, the pre-existing illnesses of each participant were not considered. However, the most relevant cause for obtaining high serum quantities may have been muscle atrophy, a common characteristic in elderly people [18,37]. This condition might have contributed to the occurrence of some type of muscle damage caused by the exercises performed, which is common in most people at advanced ages and with a high sedentary level. Even so, we cannot completely exclude the assumption that the intervention carried out regarding the EP was not totally adjusted to the participants of the study [17]. Until now, there is no strong evidence on the type of exercise that could have a positive effect on the health indicators of elderly people, especially because the ideal intensity, volume, and frequency of exercise for each person is difficult to determine since there are no specific biological markers for that [17,21].

Not all exercises are suitable for elderly people, and not all are ideal for achieving the desired muscular results. Inadequate and very intense exercises can lead to the opposite of the desired effects. However, there are some studies that say that aerobic exercise combined with resistance training improves physical performance, muscle mass, and strength in elderly people [13,14,15,21,22].

Although physical exercise alone is enough to improve the daily lives and health of older people, when combined with a balanced diet, it brings greater benefits for a healthy lifestyle [21].

## 5. Conclusions

The multidisciplinary intervention carried out made it possible to evaluate the inflammatory profile, MMPs, and TIMPs in elderly people who participated in this study. The impact of the implementation of a balanced diet with physical exercise was found especially in the increase in TNF-α and MMP-2, without changes in IL-6 and MMP-9, showing that the intervention did not cause an exacerbated inflammatory response in exercised elderly people. The intervention program implemented showed potential to contribute to better active aging strategies, taking advantage of the known benefits of exercise without inducing a harmful inflammatory response in elderly participants.

Therefore, the practice of physical exercise and a healthy diet become essential to promote the health and well-being of elderly people. However, one of the limitations we faced was the reduction in the sample size due to withdrawals because of hospital admissions and a change of day center.

In the future, it will be important to carry out more research to define the relationship between these parameters and exercise, namely, studies that focus on the most appropriate types, intensities, and frequencies of exercise, such as those that have a positive effect on the indicators of this population. Another topic that could be the object of a broader and more detailed study is the measurement, at different times, of serum levels after physical exercise. This would make it possible to assess its acute and long-term effects, considering that the best time to perform this measurement is not yet well understood; however, it is known that the concentrations of these parameters decrease in response to the adoption of a healthy diet and regular and continued practice of PA. Additionally, it would be relevant to consider the provenance of IL-6 in response to exercise (muscle or immune cells), as well as to evaluate other anti-inflammatory cytokines, such as IL-10.

## Figures and Tables

**Table 1:** Evaluation of serum levels in the control group.

CG	T0 M (±SD)	T1 M (±SD)	MD (±SD)	< *n* (%)	> *n* (%)	= *n* (%)	*p*
IL-6	0.928 (0.228)	0.892 (0.204)	0.037 (0.069)	3 (50%)	2 (33%)	1 (17%)	0.247
TNF-α	2.399 (1.192)	1.957 (1.318)	0.441 (0.987)	4 (57%)	3 (43%)	0	0.281
MMP-2	0.810 (0.278)	0.784 (0.273)	0.026 (0.103)	3 (43%)	3 (43%)	1 (14%)	0.533
MMP-9	0.937 (0.447)	1.157 (0.206)	−0.220 (0.333)	2 (29%)	5 (71%)	0	0.131
TIMP-1	1.289 (0.543)	1.457 (0.436)	−0.168 (0.244)	1 (14%)	6 (86%)	0	0.117
TIMP-2	0.603 (0.403)	0.537 (0.341)	0.066 (0.128)	4 (50%)	3 (43%)	0	0.223

Legend: CG—control group; M—mean; SD—standard deviation; MD—mean difference; *n*—sample; IL-6—interleukin 6; TNF-α—tumor necrosis factor-alpha; MMP—matrix metalloproteinase; TIMP—tissue inhibitor of metalloproteinase; T0—initial multidisciplinary diagnostic evaluation; T1—evaluation after 8 weeks; paired-sample *t*-test. All measurements are expressed in arbitrary units (AU).

**Table 2:** Evaluation of serum levels in the intervention group.

IG	T0 M (±SD)	T1 M (±SD)	MD (±SD)	< *n* (%)	> *n* (%)	= *n* (%)	*p*
IL-6	0.641 (0.129)	0.673 (0.115)	−0.032 (0.086)	5 (45.5%)	5 (45.5%)	1 (9%)	0.246
TNF-α	1.956 (0.458)	2.361 (0.450)	−0.404 (0.416)	2 (18%)	9 (82%)	0	<0.05
MMP-2	0.562 (0.092)	0.649 (0.096)	−0.087 (0.117)	2 (18%)	8 (73%)	1 (9%)	<0.05
MMP-9	0.700 (0.213)	0.726 (0.217)	−0.026 (0.115)	4 (36%)	7 (64%)	0	0.464
TIMP-1	1.159 (0.230)	1.137 (0.142)	0.022 (0.232)	5 (45.5%)	6 (54.5%)	0	0.761
TIMP-2	0.717 (0.142)	0.719 (0.085)	−0.002 (0.098)	5 (45.5%)	5 (45.5%)	1 (9%)	0.952

Legend: IG—intervention group; M—mean; SD—standard deviation; MD—mean difference; *n*—sample; IL-6—interleukin 6; TNF-α—tumor necrosis factor-alpha; MMP—matrix metalloproteinase; TIMP—tissue inhibitor of metalloproteinase; T0—initial multidisciplinary diagnostic evaluation; T1—evaluation after 8 weeks; paired-sample *t*-test. All measurements are expressed in arbitrary units (AU).

**Table 3:** Correlation between parameters.

	Evaluation Times	IG (*n* = 11)	
		rho	*p*
IL-6/TNF-α	T0	−0.182	0.593
	T1	0.337	0.311
MMP-2/TIMP-2	T0	0.689	0.019
	T1	−0.402	0.221
MMP-9/TIMP-1	T0	−0.336	0.312
	T1	−0.610	0.046

Legend: IG—intervention group; rho—Spearman’s rho; *n*—sample; IL-6—interleukin 6; TNF-α—tumor necrosis factor-alpha; MMP—matrix metalloproteinase; TIMP—tissue inhibitor of metalloproteinase; T0—initial multidisciplinary diagnostic evaluation; T1—evaluation after 8 weeks; Spearman’s rank correlation coefficient test.

## Data Availability

The raw data supporting the conclusions of this article will be made available by the authors on request.
